# Diabetic Ketoacidosis Associated With Painless Thyroiditis in a Patient Treated With an SGLT2 Inhibitor

**DOI:** 10.1155/crie/8872854

**Published:** 2025-11-21

**Authors:** Ryoichiro Aotani, Toshiaki Ohkuma, Ayaka Oshiro, Ken Okamura, Tetsuro Ago

**Affiliations:** Department of Medicine and Clinical Science, Graduate School of Medical Sciences, Kyushu University, Fukuoka, Fukuoka Prefecture, Japan

## Abstract

While sodium-glucose cotransporter 2 (SGLT2) inhibitors provide substantial benefits for glycemic control and reduced cardiovascular and renal risks, they are also associated with an increased risk of diabetic ketoacidosis (DKA) because they stimulate lipolysis. Severe thyrotoxicosis, including thyroid crisis, is a recognized cause of DKA because it promotes lipolysis and increases ketone production. Here, we report the case of a 62-year-old man with type 2 diabetes and poor glycemic control who was taking an SGLT2 inhibitor and developed DKA associated with painless thyroiditis. The blood glucose and 3-hydroxybutyric acid levels normalized after initiation of insulin therapy and fluid infusions. The thyroid function normalized without additional treatment and remained stable during follow-up. The findings in this case indicate that thyrotoxicosis may amplify the risk of DKA in the presence of the ketogenic state triggered by SGLT2 inhibitor therapy, even when the thyrotoxicosis does not reach the severity of thyroid crisis, due to the interplay with the lipolytic metabolic changes induced by thyrotoxicosis. With the increasing use of SGLT2 inhibitors, careful monitoring is essential because these medications can lower the threshold for DKA, even when triggered by minor metabolic disturbances, particularly in patients with poorly controlled glycemia.

## 1. Introduction

Sodium-glucose cotransporter 2 (SGLT2) inhibitors are a class of medications primarily used to lower blood glucose levels in patients with type 2 diabetes. They exert these effects by inhibiting glucose reabsorption in the proximal renal tubules and subsequently increasing urinary glucose excretion, independent of insulin action. Owing to their recently established cardiovascular and renal benefits in addition to their glucose-lowering effects, the prescriptions of these medications have been expanding [1], not only in patients with diabetes but also in patients with heart failure and chronic kidney disease [2–4]. While SGLT2 inhibitors provide substantial benefits, they are also associated with an increased risk of diabetic ketoacidosis (DKA) [5]. The reduction in blood glucose levels induced by SGLT2 inhibitors increases the glucagon-to-insulin ratio, thereby stimulating lipolysis and enhancing lipid oxidation while suppressing carbohydrate oxidation [6]. This metabolic shift can lead to the development of ketoacidosis without significant hyperglycemia, a condition known as euglycemic DKA [6]. Therefore, careful monitoring is essential during SGLT2 inhibitor therapy.

Severe thyrotoxicosis, including thyroid crisis, is a recognized cause of DKA [7]. Thyroid hormones stimulate lipolysis, leading to increased ketone production in the liver [7]. As most previously reported cases of DKA associated with severe thyrotoxicosis, with median free thyroxine (F-T4) levels of 68.2 pmol/L (interquartile range [IQR] 38.7–79.5; *n* = 15; one case with >99.99 pmol/L excluded) and median free triiodothyronine (F-T3) levels of 24.6 pmol/L (IQR 23.7–45.2; *n* = 6) [8], were primarily attributed to Graves' disease [8, 9], it remains unclear whether mild thyrotoxicosis, which does not progress to thyroid crisis, can lead to ketoacidosis. Considering the lipolytic effects of SGLT2 inhibitors, the synergistic interaction between thyrotoxicosis and SGLT2 inhibitors may further elevate the risk of DKA.

Here, we report a case of DKA precipitated by painless thyroiditis, which did not progress to thyroid crisis, in a patient with type 2 diabetes who was under treatment with an SGLT2 inhibitor.

## 2. Case Presentation

A 62-year-old man with a 17-year history of type 2 diabetes had been receiving treatment with metformin (2000 mg/day), gliclazide (40 mg/day), liraglutide (0.9 mg/day), and the SGLT2 inhibitor empagliflozin (25 mg/day). Empagliflozin had been prescribed for more than 3 years prior to admission. He had no prior history of thyroid disease. His HbA_1c_ levels had generally remained around 11% prior to admission. He did not exhibit anorexia and had been adhering to his prescribed medications until shortly before admission. He was admitted to the hospital for endoscopic treatment of a duodenal tumor. However, on the same day, before the procedure, he experienced vomiting. Owing to the presence of concurrent hyperglycemia (17.2 mmol/L), he was referred to the diabetes department for further evaluation. There had been no prolonged fasting or dietary restriction prior to admission, and empagliflozin had not been withheld prior to the planned endoscopic procedure.

On admission, his height was 155.0 cm and weight was 56.8 kg, corresponding to a BMI of 23.6 kg/m^2^, with no recent or sudden changes in weight. He was afebrile, and his heart rate was regular at 95 beats/min. The thyroid gland was normal in size and consistency, with no pain or tenderness. His HbA_1c_ level was 10.1%. Laboratory test results showed an arterial blood pH of 7.33 and an HCO_3_^−^ level of 12.3 mmol/L, consistent with high anion gap metabolic acidosis (27.2 mmol/L) (Table 1). Serum lactate was 1.4 mmol/L, within the normal range. Urinary ketone bodies (2+) were also observed, confirming the presence of ketoacidosis. No arterial or venous blood gas analysis had been available prior to admission. The C-peptide level was elevated, indicating preserved endogenous insulin secretion. There were no symptoms indicative of thyrotoxicosis. However, the serum F-T4 level was elevated (29.61 pmol/L), and the thyroid-stimulating hormone (TSH) level was suppressed (0.06 mIU/L), although the serum F-T3 level remained within the normal limits (3.98 pmol/L). TSH receptor antibody, thyroglobulin antibody, and antithyroid peroxidase antibody were all negative, suggesting an episode of painless thyroiditis associated with nonthyroidal illness. Ultrasound examination of the thyroid gland revealed no abnormalities in morphology or echogenicity, no increased intraglandular blood flow, and no evidence of a nodule.

After discontinuing all oral hypoglycemic medications and initiating subcutaneous insulin injections along with extracellular fluid infusions, the blood glucose and 3-HBA levels decreased to their normal ranges and stabilized, and urinary ketone bodies became negative within 3 days. On Day 10, oral semaglutide (3 mg) and empagliflozin (10 mg) were resumed, and insulin therapy was gradually tapered.

The patient was discharged with 12 U of premixed insulin on Day 21. By Day 15, the F-T4 and TSH levels had spontaneously normalized without antithyroid drug administration (20.34 pmol/L and 0.63 mIU/L, respectively). These levels remained within the normal ranges until follow-up on Day 64, confirming the diagnosis of painless thyroiditis.

## 3. Discussion

In the present case, a patient with type 2 diabetes who was taking an SGLT2 inhibitor experienced DKA associated with thyrotoxicosis induced by painless thyroiditis, which did not progress to thyroid crisis. The blood glucose and 3-HBA levels normalized after initiation of insulin therapy and fluid infusions. The thyroid function normalized without additional treatment and remained stable during follow-up. This case highlights the potential for relatively mild thyrotoxicosis, which does not reach the severity of thyroid crisis, to precipitate DKA in patients under treatment with SGLT2 inhibitors.

Thyrotoxicosis, particularly thyroid crisis, is an established trigger for DKA, primarily due to the glycogenic and lipolytic effects of excessive levels of thyroid hormones, which subsequently elevate the serum ketone level [7, 10]. An ~10-fold increase in β-adrenergic sensitivity for lipolysis has been reported in hyperthyroid patients [11]. Meanwhile, thyrotoxicosis is known to increase insulin degradation and glucagon secretion [12], both of which contribute to the development of DKA. Graves' disease is the most commonly identified underlying condition in such cases [8, 9]. DKA has also been reported in hyperthyroid patients with normal T3 levels [13, 14] and in a patient with Hashitoxicosis [15].

In contrast to the previously reported cases, this is the first documented case of a patient with type 2 diabetes who was taking an SGLT2 inhibitor and developed DKA where thyrotoxicosis associated with painless thyroiditis, which remained mild and did not escalate to thyroid crisis, may have contributed as a possible trigger in the presence of other precipitating factors. We speculate that the development of DKA in the present patient may have been fundamentally attributed to the lipogenic effects of the SGLT2 inhibitor combined with poor glycemic control. SGLT2 inhibitors lower the threshold for renal glucose excretion, mimicking starvation and increasing ketone production and reabsorption [5]. The interplay between the lipolytic metabolic changes induced by thyrotoxicosis and the ketogenic state triggered by SGLT2 inhibitor use may have amplified the risk of DKA, even in the absence of thyroid crisis. Although fasting associated with endoscopic procedures can be a potential contributor to SGLT2 inhibitor–related DKA, in the present case, there had been no prolonged fasting or dietary restriction prior to admission, and therefore this factor was unlikely to have played a role. On the other hand, although ketoacidosis was present, as supported by urinary ketone bodies (2+), we cannot exclude the possibility that underlying renal dysfunction may have contributed to the acidosis, since no blood gas analysis had been performed prior to admission.

DKA can occur in patients under treatment with SGLT2 inhibitors, even in the presence of uncharacteristically mild to moderate glucose elevations [6], as observed in the present case (blood glucose level: 17.2 mmol/L). This atypical presentation may delay the testing and detection of ketoacidosis. Notably, two cases of euglycemic DKA associated with thyrotoxicosis in patients under SGLT2 inhibitor treatment have been reported [16]. Therefore, when patients who are taking SGLT2 inhibitors feel unwell, euglycemic DKA should be considered, and prompt monitoring of ketonuria or ketonemia is recommended [6].

Recent guidelines advocate for the use of SGLT2 inhibitors in the management of patients with heart failure and chronic kidney disease, irrespective of the presence of diabetes [2–4]. Consequently, the incidence of DKA is anticipated to increase, particularly given a recent report describing the development of DKA in patients without diabetes who were under treatment with SGLT2 inhibitors [17]. It is important to recognize that even minor triggers can cause DKA in patients who are taking SGLT2 inhibitors, underscoring the importance of appropriate ketone testing regardless of the blood glucose level or symptom severity.

In conclusion, we have reported the case of a patient with type 2 diabetes and poor glycemic control who was taking an SGLT2 inhibitor and developed DKA associated with painless thyroiditis. With the increasing use of SGLT2 inhibitors, careful monitoring is essential, because these medications can lower the threshold for DKA, even when triggered by minor metabolic disturbances, particularly in patients with poorly controlled glycemia.

## Figures and Tables

**Table 1:** Laboratory measurements on admission.

Parameters	Results	Normal range
Glucose parameters		
Glucose	17.2 mmol/L	4.1–6.1 mmol/L
HbA_1c_	10.1%	4.9%–6.0%
C-peptide	2.88 nmol/L	0.25–1.05 nmol/L
Anti-GAD antibody	<5.0 U/mL	<5.0 U/mL
Arterial blood gas analysis		
pH	7.33	7.35–7.45
HCO_3_^−^	12.3 mmol/L	20.0–24.0 mmol/L
Anion gap	27.2 mmol/L	10.0–14.0 mmol/L
Lactate	1.4 mmol/L	0.5–2.2 mmol/L
Serum ketone bodies		
Acetoacetate	186 μmol/L	0–55 μmol/L
3-HBA^a^	841 μmol/L	0–85 μmol/L
Thyroid function		
TSH	0.06 mIU/L	0.61–4.23 mIU/L
Free T3	3.98 pmol/L	3.38–6.76 pmol/L
Free T4	29.61 pmol/L	12.87–23.17 pmol/L
TRAb	<0.8 IU/L	<2.0 IU/L
TgAb	14.6 IU/mL	<30.0 IU/mL
TPOAb	16.0 IU/mL	<30.0 IU/mL
Renal function		
eGFR	22.2 mL/min/1.73 m^2^	>60.0 mL/min/1.73 m^2^
Urinalysis		
Protein	3+	
Sugar	4+	
Ketone body	2+	

*Note:* TgAb, anti-thyroglobulin antibody; TPOAb, anti-thyroid peroxidase antibody; HbA1c, glycated hemoglobin; T4, thyroxine; T3, triiodothyronine; TRAb, TSH receptor antibody.

Abbreviations: eGFR, estimated glomerular filtration rate; GAD, glutamic acid decarboxylase; HBA, hydroxybutyric acid; TSH, thyroid-stimulating hormone.

^a^The 3-HBA level was measured at 36 hours after insulin administration.

## Data Availability

Data sharing are not applicable to this article as no datasets were generated or analyzed during the current study.
